# A N*arcissus mosaic* viral vector system for protein expression and flavonoid production

**DOI:** 10.1186/1746-4811-9-28

**Published:** 2013-07-13

**Authors:** Huaibi Zhang, Lei Wang, Donald Hunter, Charlotte Voogd, Nigel Joyce, Kevin Davies

**Affiliations:** 1 The New Zealand Institute for Plant & Food Research Limited (PFR), Private Bag 11600 Palmerston North, New Zealand; 2 PFR, Private Bag Private Bag 92169, Auckland 1142 New Zealand; 3 PFR, Private Bag 4704 Christchurch, New Zealand

## Abstract

**Background:**

With the explosive numbers of sequences generated by next generation sequencing, the demand for high throughput screening to understand gene function has grown. Plant viral vectors have been widely used as tools in down-regulating plant gene expression. However, plant viral vectors can also express proteins in a very efficient manner and, therefore, can also serve as a valuable tool for characterizing proteins and their functions in metabolic pathways *in planta*.

**Results:**

In this study, we have developed a Gateway®-based high throughput viral vector cloning system from *Narcissus Mosaic Virus* (NMV). Using the reporter genes of GFP and GUS, and the plant genes *PAP1* (an R2R3 MYB which activates the anthocyanin pathway) and selenium-binding protein 1 (SeBP), we show that NMV vectors and the model plant *Nicotiana benthamiana* can be used for efficient protein expression, protein subcellular localization and secondary metabolite production.

**Conclusions:**

Our results suggest that not only can the plant viral vector system be employed for protein work but also can potentially be amenable to producing valuable secondary metabolites on a large scale, as the system does not require plant regeneration from seed or calli, which are stages where certain secondary metabolites can interfere with development.

## Introduction

Characterizing the functions of proteins encoded by newly identified DNA sequences is a fundamental aspect of molecular research. Aspects of interest may include the sub-cellular localization of the protein, the activity of the purified protein, or the phenotype resulting from its over-production *in planta*. The demand for systems to screen for gene function has grown with the increased availability of candidate sequences from next generation sequencing transcriptomics studies and the completion of whole genome sequences. Functional studies of large numbers of genes using stable plant transformation is time-consuming and costly, and thus there is a need for high-throughput screening systems [1]. Preferably, such systems would allow for both high-level production of the encoded protein and observation of the effect on plant phenotype. Systems that enable gene introduction into mature plants have a further advantage of allowing production of proteins or metabolites that may inhibit the usual plant transformation and regeneration processes.

*Agrobacterium*-mediated transient protein expression systems using conventional binary vectors have been developed to perform various screening tasks [1-3]. Transient expression using plant viral expression vectors can over-express genes not only locally but also systemically before virus-induced gene silencing takes over in the plant tissue. This systemic infection enables production of much larger quantities of plant material in a relative short time frame. Therefore, a viral expression system, combined with the appropriate host plant, could provide an efficient tool for studying protein functions and producing valuable secondary metabolites *in planta* without having to generate stable transgenic plants. While plant viral vectors have been widely used to down-regulate the expression of endogenous genes [4-9], they have been also developed to over-express heterologous proteins [3,10-16]. However, there can be limitations associated with the different viral systems used.

For example, gene silencing mediated through *Potato virus X* (PVX) is superimposed upon a visually observed response to the virus itself [17], *Tobacco mosaic virus* (TMV) can cause pathogenic responses in the host, and it can be difficult to prevent the spread of TMV to nearby non-target plants.

*Narcissus Mosaic Virus* (NMV) is a potexvirus with a single-stranded RNA genome. Infection of *Nicotiana benthamiana* plants with NMV (GenBank AY225449) does not cause clearly visible symptoms in the vegetative organs. Plant growth and development in daffodils (*Narcissus pseudonarcissus*) were apparently normal post NMV infection [18]. *N. benthamiana* has become one of the standard models for plant biology, including high-throughput over-expression studies [19-22]. The development of a NMV-based expression system for this species would therefore provide an excellent research and biotechnology tool. It may be particularly useful for studies on the anthocyanin biosynthetic pathway, as the *N. benthamiana* varieties used in most laboratories lack anthocyanin production. The anthocyanin biosynthetic pathway, as part of the larger flavonoid pathway, is an important model for a wide range of plant studies, because of both its advantages as a visible reporter system and its key roles in diverse plant functions [23-25]. It is involved in processes such as pollination, fruit dispersal, pathogen resistance and environmental stress tolerance, has been a key model for elucidating transcriptional regulation in plants, and there is growing evidence for the beneficial effects on human health of dietary flavonoids [24,26-28]. In the study presented here, the potential of NMV and *N. benthamiana* as a protein expression system and for secondary metabolite production are explored using both characterized reporter genes (GFP and GUS) and the flavonoid pathway as models. Taking advantage of the many previous studies on transcriptional regulation of flavonoid biosynthesis, a well-characterized R2R3 MYB transcription factor AtMYB75 (PAP1) that promotes anthocyanin biosynthesis in *Arabidopsis* was used to induce significant changes in metabolite production in inoculated plants, including visible pigment production.

### Experimental procedures

#### Plant materials

*Nicotiana benthamiana* plants were grown during the southern hemisphere autumn in containment rooms of a double-skin plastic greenhouse facility in Palmerston North, New Zealand (40°21’00”S; 175°36'36”E). The temperature was controlled by air conditioners to 22 +/−2°C, and no additional lighting was supplied. Fully opened leaves from plants with approximately 10 fully opened leaves were used for biolistic bombardments of viral vectors onto detached leaves and viral inoculation on attached leaves.

#### Viral vector construction

**Figure 1:**
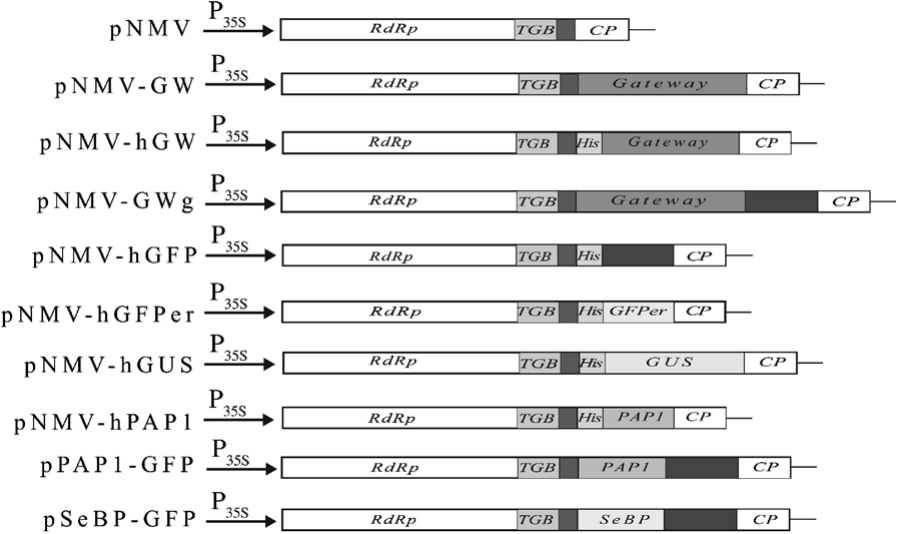
**The DNA regions of the NMV vectors cloned into pCass2 between the StuI and BamH1cloning sites.** RdRp: RNA-dependent RNA polymerase; TGB: Triple gene block proteins; Ps: subgenomic promoter duplicate for coat protein; GW: Gateway cloning cassette; His: polyhistidine tag, 6×histidine; PAP1: *Arabidopsis* PAP1; SeBP: *Arabidopsis* selenium-binding protein; GFP: Green fluorescence protein; GFPer: Green fluorescence protein with intracellular targeting sequences to endoplasmic reticulum; GUS: GUS sequence without intron; CP: coat protein.

A DNA sequence encoding a full length genomic RNA of *Narcissus Mosaic Virus* New Zealand strain (GenBank: AY225449) was cloned into pCass2 [29] between StuI and BamHI sites to generate pNMV (Figure 1). In pNMV, a duplicated subgenomic promoter for NMV coat protein and AvrII/ApaI cloning sites behind the duplicated subgenomic promoter were created in front of the native coat protein subgenomic sequence. To make a high-throughput cloning vector, the Gateway® cloning site (Invitrogen, Auckland, New Zealand) was transferred into pNMV. The attR1-CmR-ccdB-attR2 Gateway® cassette was amplified using the primers 5′-atcctaggACAAGTTTGTACAAAAAAGCAGGCT -3′ (forward) and 5′-tacctaggGGACCACTTTGTACAAGAAAGCTGGGA -3′ (reverse) and a high fidelity DNA polymerase PWO (Roche, Auckland, New Zealand). The AvrII flanked attR1-CmR-ccdB-attR2 cassette was inserted into the AvrII site of pNMV to generate the destination vector pNMV-GW (Figure 1).

To facilitate purification of the proteins expressed using pNMV-GW, a His-tag coding sequence was placed in front of the Gateway® cassette. An AvrII-6×His- attR1-CmR-ccdB-attR2-ApaI cassette was assembled using overlapping PCRs, with the first set of primers being 5′- CATCATCACCATCACCATGGACAAGTTTGTACAAAAAAGCAG-3′ (forward) and 5′-atcgatgggcccACCACTTTGTACAAGAAAGCTG-3′ (reverse), which generated 6×His-attR1-CmR-ccdB-attR2, and the second set of primers being 5′-atcctaggATGCATCATCACCATCACCATGGA-3′ (forward) and 5′-atcgatgggcccACCACTTTGTACAAGAAAGCTG -3′ (reverse), which added AvrII and ApaI sites to the 5′ and 3′ ends of the cassette, respectively. The generated AvrII-6×His- attR1-CmR-ccdB-attR2-ApaI cassette was then inserted between the AvrII and ApaI sites of pNMV to generate the destination vector pNMV-hGW (Figure 1).

To enable GFP tagging of proteins, a vector containing a GFP tag next to the cloning cassette was also constructed. The GFP coding sequence was derived from the *gfp5* sequence [30] by cloning it attached to the NMV sequence using the same cloning procedures as used for construction of pNMV, but using the primer pair 5′-atcctaggACAAGTTTGTACAAAAAAGCAGGCT -3′ (forward) and 5′-atcgatgggcccACCACTTTGTACAAGAAAGCTG -3′ (reverse) . The AvrII-attR1-CmR-ccdB-attR2-ApaI Gateway® cassette was subsequently inserted in front of the GFP sequence using the AvrII/ApaI sites such that any sequence inserted into the vector encoded a protein fusion in frame with GFP attached at the C-terminus of the expressed protein of interest. This destination vector was named pNMV-GWg (Figure 1).

The test proteins used were GFP, GFP-ER containing intracellular targeting sequences to endoplasmic reticulum [30], β-glucuronidase (GUS), *Arabidopsis* Production of Anthocyanin Pigment 1 (PAP1) and *Arabidopsis* Selenium-Binding Protein (SeBP). The coding sequences of these proteins were obtained using PCR-amplification with the high fidelity DNA polymerase PWO and the following template DNAs –*pBIN-m-gfp5-ER*[30] for *GFP* and *GFP-ER; pRT99-GUS for GUS*; PAP1, pGreenII-cPAP (provided by Dr Roger Hellens, Plant & Food Research, New Zealand); SeBP, pGreenII-cPAP (provided by Dr Roger Hellens, Plant & Food Research, New Zealand) for PAP1, *Arabidopsis* cDNA for SeBP (based on the sequence from Agalou et al. (2005). The primers used are listed in Supplementary Data (Additional file 1: Table S1). Through BP and LR reactions as described in the manufacturer’s Gateway® protocols (Invitrogen), the PCR products flanked with attB1 and attB2 were then cloned into pNMV-hGW to form the expression vectors pNMV-hGFP, pNMV-hGFPer, pNMV-hGUS and pNMV-hPAP1, and PAP1 and SeBP coding sequences into pNMV-GWg to form the subcellular localization vectors pPAP1-GFP and pSeBP-GFP.

#### Biolistic introduction of viral vectors into N. benthamiana leaves

The pCass2-based viral vector DNAs were propagated in *Escherichia coli*, extracted, and purified using the Plasmid Plus Maxi Kit following the manufacturer’s instructions (QIAGEN, Bio-Strategy Ltd, Auckland, New Zealand). DNA-coated gold particles (1 μm) were delivered into the cells of detached fully opened *N. benthamiana* leaves using a helium-driven particle inflow gun [31] and the method of Shang and co-workers [32]. Each detached leaf was bombarded once with 8.0 μL of DNA/gold suspension and subsequently placed in a Petri dish with a small amount of water to prevent dehydration, and placed in a culture room in the dark at 25°C for 4 to 5 days, and subsequently stored in a −80°C freezer until required.

#### Inoculation of viral particles into the leaves of intact *N. benthamiana* plants

The biolistically transformed detached leaves, being stored in the −80°C freezer, were sealed in small plastic bags with a small amount of water and crushed with a roller to obtain leaf saps. The leaf saps obtained from the detached leaves that had been transformed with different viral vector DNAs were respectively applied by rubbing onto fully-opened carborundum-dusted leaves of the greenhouse plants that had a total of 6–10 leaves. The greenhouse plants were monitored visually and microscopically for the symptoms.

#### Subcellular localization of proteins with GFP tagging

GFP fluorescence of the leaves bombarded with pNMV-hGFP, pNMV-hGFPer, pPAP1-GFP or pSeBP-GFP was monitored regularly during the incubation period using a fluorescent microscope (Olympus SZX), and final digital images (after 4–5 days) captured using a Leica TCS SP5 confocal microscope (located at Massey University, Palmerston North, New Zealand).

#### Protein extraction, purification and western analysis

The GFP protein expression in the leaves of the intact plant was monitored using UV light (Black Ray B100-AP lamp, UV products, Upland, CA 91786, USA) and the leaves that showed systemic infection according to GFP fluorescence were collected for protein extraction.

For the isolation of the proteins containing the His-tag, leaves (0.5-0.7 g) were ground to a fine powder under liquid nitrogen using a mortar and pestle. Subsequently, the total proteins were extracted by adding 3.0 mL of the Yeast Protein Extraction Reagent Y-PER®-S (Pierce, USA), 30 μL of tris(hydroxypropyl)phosphine and 10.0 μL of Halt Protease Inhibitor (Pierce, USA). The supernatant (12000 g for 15 min) containing the total soluble proteins was mixed with 1.0 mL Ni-charged Chelating Sepharose to bind His-tagged proteins. Ni-charged Chelating Sepharose was prepared and the centrifugation approach of Ni-affinity purification was employed according to the manufacturer’s protocol (Chelating Sepharose Fast Flow, Amersham Biosciences, Auckland, New Zealand).

For concentrating GUS protein, the total proteins were extracted using the extraction buffer: 50 mM of phosphate buffer (pH 7.0) containing 5 mM of DTT, 2 mM of EDTA, 0.1% SDS and 0.1% Triton X-100. Subsequently, the supernatant was applied to a 3 mL of open column of diethylaminoethyl cellulose (DEAE) Sepharose (Sigma). The DEAE column was prewashed with the protein extraction buffer prior to sample loading. The loaded column was washed with two column volumes of 0.05 M and then 0.1 M NaCl. One-mL fractions were subsequently collected by eluting the column with 0.2 M NaCl. The concentration of GUS protein in each fraction was monitored through the blue color generated by GUS enzyme activity, by incubating 5.0 μL of the fractions in 60.0 μL of X-gluc staining buffer [32] for 2 min at 37°C. Subsequently, the affinity-purified and the DEAE-concentrated protein fractions were all subjected to PAGE and Western blot analysis.

Sample separation for Western blot analysis of the protein samples was carried out using precast 4-20% (w/v) SDS-polyacrylamide (SDS-PAGE) gels and the manufacturer’s protocol (iGELs, Gradipore, USA). Protein samples were separated on two identical gels, with Precision Plus Protein All Blue Standards (Bio-Rad, Auckland, New Zealand) as molecular mass markers. After separation, one of the gels was stained with Coomassie Blue and the other gel blotted onto a polyvinyllidene difluoride (PVDF) membrane (Millipore), with transfer assisted using an electro-eluter (model 422, Bio-Rad). The PVDF blots were probed with Anti-β-Glucuronidase Rabbit IgG fraction (Molecular Probes, Oregon, USA), Anti-GFP rabbit IgG fraction (Molecular Probes, Oregon, USA) or mouse monoclonal His-Tag antibody (Novagen, Madison, WI, USA). Western colorimetric detection was carried out using goat Anti-Rabbit IgG –Alkaline Phosphate (AP) (Sigma) or goat anti-mouse AP-conjugated IgG Fab fragments (Boehringer-Mannheim GmbH) secondary antibodies. All the antibodies were used according to the respective instructions.

#### Flavonoid analysis

The studies on the effect of pNMV-hPAP, pPAP1-GFP and pNMV-GFP on flavonoid production in *N. benthamiana* were carried out by inoculating fully opened leaves of plants.

Thin-layer chromatography (TLC) was carried out to confirm the presence of anthocyanins in leaves showing colored foci. Leaves were crushed in water as described for protein extraction, and the extract hydrolyzed with 3N HCl for 45 min to remove any sugar residues from the anthocyanins (yielding anthocyanin aglycones). The aglycones were enriched in amyl alcohol, spotted onto cellulose TLC plates (CE-400, POLYGRAM® polyester sheets, MACHEREY-NAGEL, Germany), and separated on TLC plates using formic acid/concentrated HC/water solvent (5:2:3, v:v:v).

Flavonoids present in the leaf extracts were analysed by LC-Mass Spectroscopy (LCMS), fitted with an LCMS ion-trap (LTQ ion-trap, Thermo Finnigan, San Jose, California) and electro-spray ionization (ESI) in the negative and positive ion modes. Single MS, MS2 and MS3 data were collected based on parent masses from 280–2000 m/z after elution via a PDA detector scanning 220–600 nm (UV-visible). Aliquots of 5 μl of sample were separated on a Synergi-Hydro RP, 4 _m, 250 × 2.1 mm column with 4 × 2 mm guard cartridge (Phenomenex Ltd) at a temperature of 25°C with a mobile phase flow rate of 300 μL/min. The mobile phase consisted of water (A) and acetonitrile (B) both containing 1% formic acid (FA) with gradient elution from 98% A to 50% B over 45 minutes for general qualitative screening. The column was then washed to a concentration of 80% (B) for 5 minutes and then re-equilibrated with 98% (A).

#### Northern RNA analysis

Total RNA was extracted from the leaves using Trizol reagent according to the manufacturer’s instruction (Invitrogen). Twenty μg of total RNAs were resolved in a 1.2% agarose gel containing 0.66 M of formamide. After electrophoresis, RNAs were blotted onto nylon membranes (Hybond N^+^ membrane, GE Healthcare) and cross-linked to the membrane by UV-C at 70 000 μJ cm^-2^ (Hoefer, San Francisco, CA) for 1 min. Radiolabeled ([α-^32^P]dCTP) was used to prepare [^32^P]-radiolabeled DNA probes using the HighPrime (Roche Applied Science) labelling kit, with GFP and PAP1 DNAs being used as templates. Hybridization against [^32^P]-radiolabeled DNA probes was carried out as described in Deroles et al. (1998), except with a final wash stringency of 65°C and 0.5 × SSC to remove nonspecific bindings of the radioactive signals.

## Results

### Construction of NMV expression vectors

To enable NMV infection in plants without having to use *in vitro* reverse transcription, a construct was prepared that placed a DNA sequence for the viral RNA genome in a plant expression cassette, driven by the 35SCaMV promoter, with a duplicate subgenomic promoter for the coat protein of NMV placed in front of a cloning site immediately upstream of the native sequence for the coat protein (pNMV, Figure 1). To allow for high throughput cloning, a version of pNMV was prepared that utilizes the Gateway® site-specific recombination system between the duplicate subgenomic promoter and the native subgenomic promoter for the coat protein for rapid cloning, specifically the (pNMV-GW, Figure 1). The pNMV-GW vector was constructed so that no start codon occurred in front of the Gateway® cassette, enabling the endogenous start codon of any coding sequence (CDS) to be used.

The pNMV-GW vector was further modified to facilitate intracellular localization of proteins and rapid protein purification. For localization studies pNMV-GW was modified to result in formation of the expressed proteins with Green Fluorescent Protein (GFP) as a C-terminal fusion of a expressed protein (pNMV-GWg, Figure 1). The GFP CDS was placed downstream of the Gateway® attB2 recombination sequence for easier in-frame fusion of the target CDS and GFP. Target sequences for expression are PCR amplified, spanning from the CDS ATG to the codon before the stop codon, using primers flanked with the Gateway® attB1 and attB2 sequences.

To allow purification of expressed proteins using Ni-based affinity purification, a leader sequence containing a six-histidine peptide tag and an ATG start codon was placed in frame with attB1, creating pNMV-hGW (Figure 1), and providing for Gateway® cloning into the attB1 and attB2 vector sequences.

### Subcellular localization of proteins expressed with NMV vectors

Two different versions of GFP were expressed using pNMV-hGW to examine the affect on protein sub-cellular localization of the inclusion of either the His-tag or two ER-localization signals used to aid protein accumulation. The basic chitinase signal peptide and the ER-retention signals were used, the *Arabidopsis* basic chitinase signal on the N-terminus and the HDEL motif [30] on the C-terminus, giving pNMV-hGFPer (Figure 1). The basic chitinase signal was placed immediately following the His tag.

**Figure 2:**
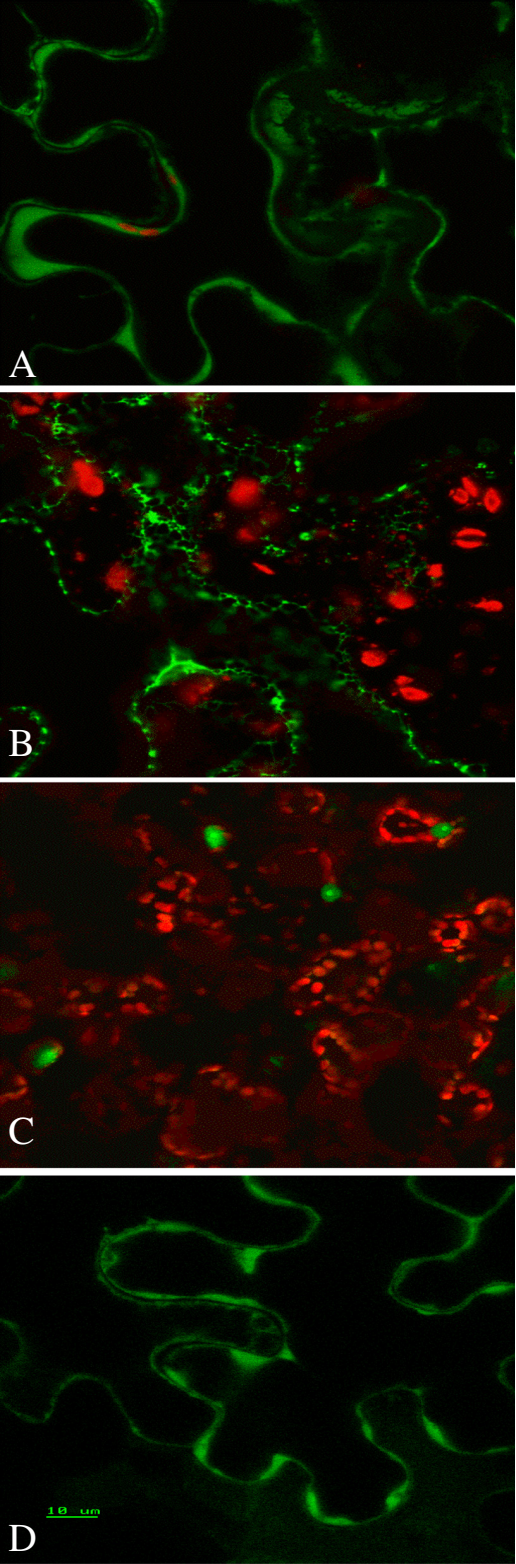
**Subcellular localization of GFP and GFP-tagged proteins expressed in the leaves of *N. benthamiana*. A**: GFP without basic chitinase signal peptide at the N-terminus and the ER-retention peptide HDEL at the C-terminus; **B**: GFP containing the basic chitinase signal peptide at the N-terminus and the ER-retention peptide HDEL at the C-terminus; **C**: PAP1-GFP fusion protein; **D**: SeBP-GFP fusion protein.

The two vectors were introduced into *N. benthamiana* leaves using biolistics and the sub-cellular pattern of accumulation determined using confocal microscopy. The His-tag at the N-terminus did not alter the typical intracellular distribution of GFP signal to the cytoplasm (Figure 2A). However, the ER-localization signals caused GFP signal to accumulate in a pattern that probably represents the ER lumen (Figure 2B). The presence of the His-tag apparently did not affect the action of the ER-localization signals.

Two *Arabidopsis* genes, *PAP1* (AtMYB75, 28 kDa) and selenium-binding protein 1 (*SeBP1*, 55 kDa), were also used in the localization study. PAP1 is a relatively small R2R3 MYB transcription factor, the over-expression of which has been demonstrated to induce anthocyanin biosynthesis in *Arabidopsis* and tobacco [33]. SeBP1 is a much larger protein that has been extensively characterized for its role in heavy metal tolerance [34]. The GFP-tagging vector pNMV-GWg was used to generate pPAP1-GFP and pSeBP-GFP (Figure 1). After biolistic transformation of the detached *N. benthamiana* leaves with pPAP1-GFP and pSeBP-GFP DNAs, localization of the GFP-tagged PAP1 and SeBP1 proteins was examined using confocal microscopy. Nuclear localized GFP signal was observed for pPAP1-GFP (Figure 2C) and a cytoplasmic GFP signal for pSeBP-GFP (Figure 2D), matching the known localization of the native proteins.

### Protein expression and purification using the NMV vectors

**Figure 3:**
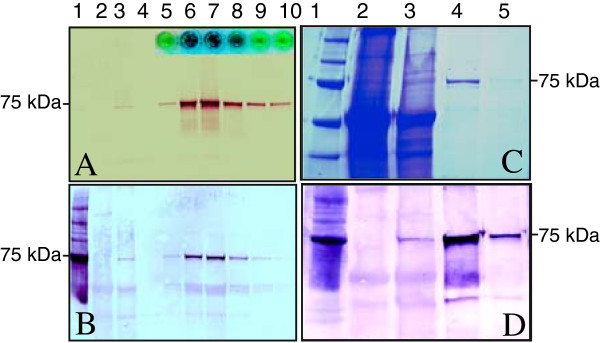
**Expression of His-tagged GUS protein (69.4 kDa) in the leaves of *N. benthamiana *with NMV viral vector pNMV-hGUS. A**: GUS protein was extracted and separated on a 4-20% gradient SDS-PAGE gel and transferred onto PVDF membrane and was probed with Anti-β-Glucuronidase Rabbit IgG as the primary antibody; the insert wells showing blue color indicate the strength of GUS activity in each fraction. **B**: The identical PAGE gel as in A was used and probed with mouse monoclonal His-Tag antibody. **C**: GUS protein purified with Ni-Affinity Sepharose, separated on 4–20% SDS-PAGE gel was visualized with Coomassie Blue; **D**: An identical gel was used for the Western analysis and probed with mouse monoclonal His-Tag antibody as the primary antibody. For C and D, Lane1 Protein Marker; Lane 2: Mock Leaves; Lane 3, total protein from the leaves inoculated with pNMV-hGUS; Lane 4, the first elution of GUS from the Ni-affinity Sepharose; Lane 5–10, fractions of the GUS elution from the Ni-affinity Sepharose.

To examine the flexibility of the NMV system, production of not only the relatively small reporter GFP (approximately 27 KDa), but also that of the relatively large reporter protein GUS (approximately 68 KDa) were examined, using the vector pNMV-hGUS. GUS activity, as assayed by GUS staining, was evident five days after biolistic bombardment of pNMV-hGUS into *N. benthamiana* leaves (Additional file 2: Figure S1). GUS protein accumulation was confirmed in leaves of intact plants in the greenhouse that had been inoculated with the extracts of the pNMV-hGUS-bombarded leaves (Additional file 2: Figure S1), using a activity assay, coomassie Blue protein staining and the Western blot analysis (Figure 3). Total protein extracted from the inoculated greenhouse plants was passed through an open DEAE column and the GUS activity assayed in the eluted fractions. Strong blue color was observed within 2 min of incubation only in the fractions that contained enriched GUS protein (Figure 3A, insert). Western analysis using anti-GUS (Figure 3A) and anti-His antibody (Figure 3B) detected GUS protein in those DEAE column fractions that had shown high GUS activity.

The pNMV-hGUS plants were also tested for their applicability with the rapid Ni-affinity purification system. Commassie Blue staining (Figure 3C) and Western analysis (Figure 3D) showed that the Ni-affinity protocol was highly effective for purification of the GUS protein expressed from pNMV-hGUS-inoculated greenhouse plants. The GUS protein purified with Ni-affinity was equivalent to 10 mg per kg fresh weight of the leaf shown in Additional file 2: Figure S1.

**Figure 4:**
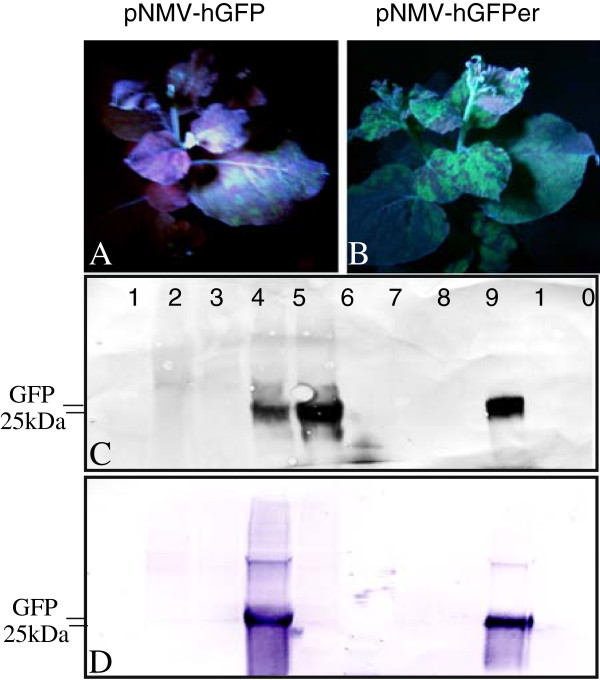
**Expression of His-tagged GFP (27 kDa) in the leaves of *N. benthamiana *with NMV viral vector pNMV-GFP and pNMV-GFPer. A**: Image taken under UV light, showing systemic distribution of GFP without basic chitinase signal peptide at the N-terminus and the ER-retention peptide HDEL at the C-terminus; **B**: Image taken under UV light, showing systemic distribution of GFP containing basic chitinase signal peptide at the N-terminus and the ER-retention peptide HDEL at the C-terminus; **C**: total proteins were extracted from the leaves inoculated with pNMV-GFP and pNMV-GFPer respectively and separated on a 4-20% SDS-PAGE gel, transferred onto PVDF membrane and probed with Anti-GFP rabbit IgG. Lane 1: Protein Marker; Lane 2: Mock control; Lane 3: leaves inoculated with pNMV-PAP1, Lane 4: inoculated with pNMV-hGFP; Lane 5: the leaves inoculated with pNMV-hGFPer; Lane 6–10 were the same samples as in Lane 1–5 but having gone through Ni-Sepharose affinity purification. **D**: Identical Western blot as in C but probed with mouse monoclonal His-Tag antibody as the primary antibody.

The use of GFP as one of the reporters also allowed for easy examination of the timeframe of NMV infection and spread. Local infection foci were apparent about five days post inoculation (dpi) and systemic infection was well established by about 10 dpi. The results were similar whether pNMV-hGFP or pNMV-hGFPer was used. However, long-term accumulation of GFP appeared stronger for pNMV-hGFPer than pNMV-hGFP (Figures 4A and B). Western analysis of total protein extracts showed that anti-GFP antibody detected GFP accumulation from both pNMV-hGFPer and pNMV-hGFP (Figure 4C), but anti-His antibody detected GFP accumulation only from pNMV-hGFP (Figures 4B and C). When the total proteins were purified using the Ni-affinity method, strong signals were detected on the Western analysis with both the anti-GFP and anti-His-antibodies with samples from plants inoculated with pNMV-hGFP, but not with those from pNMV-hGFPer plants (Figures 4B and C).

### Expression of PAP1 in *N. benthamiana* using pNMV vector

The GFP and GUS protein expression experiments demonstrate that NMV is an effective tool for over-expressing proteins in *N. benthamiana*. To examine its utility for proteins of biological interest and application for studying metabolic pathways, an *Arabidopsis* protein was tested, PAP1 (AtMYB75, 28 kDa). The PAP1 CDS was cloned into pNMV-hGW to generate pNMV-hPAP1 (Figure 1).

**Figure 5:**
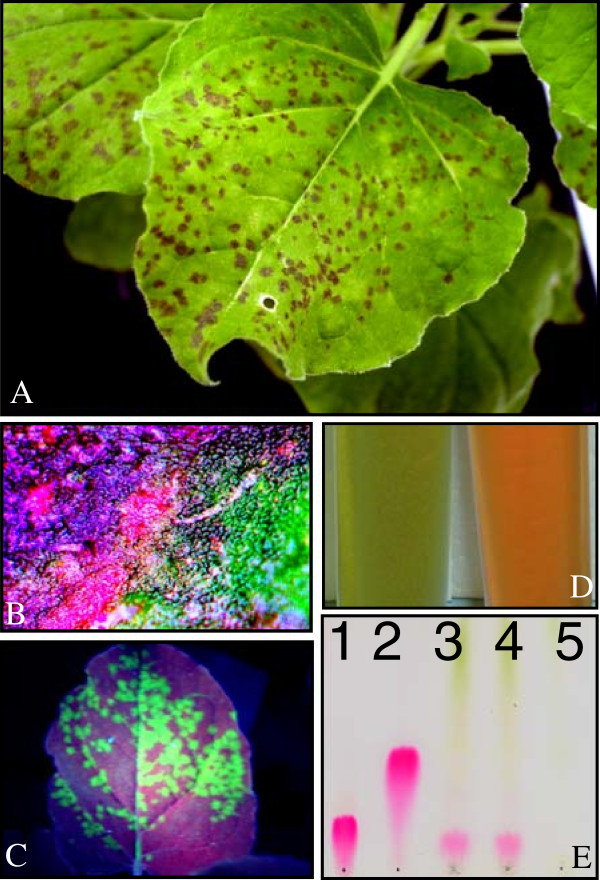
**Expression of PAP1 in the leaves of *N. benthamiana *with pNMV-hPAP1 viral vector. A**: Anthocyanic foci at 7 dpi; **B**: Uneven-colored anthocyanic pigmentation across foci; **C**: GFP foci; **D**: Showing color difference of the extract supernatants from leaves inoculated with pNMV-hGFP (L) and pNMV-hPAP1 (R). **E**: TLC displaying anthocyanidin extracted from the leaves inoculated with pNMV-hPAP1 vector, from L to R: delphinidin, cyanindin, sample1, sample 2 and pNMV-hGFP control.

The vectors pNMV-hPAP1 along with the control pNMV-hGFP, were biolistically introduced into detached *N. benthamiana* leaves. After five days in the dark, the leaves that received pNMV-hPAP1 DNA produced dark-colored foci, while no colored foci were visible on pNMV-hGFP leaves (Additional file 3: Figure S2). Extracts of leaves of these plants were then inoculated onto fully opened leaves of *N. benthamiana* greenhouse plants, while control plants were mock-inoculated. Large numbers of dark-red foci appeared in the leaves of plants treated with pNMV-hPAP1 inoculate after 7 dpi (Figure 5A). The pigmentation was uneven across the colored foci (Figure 5B), as might be expected from the nature of the viral infection process. The pNMV-hGFP inoculate did not generate visible foci, although GFP foci were apparent under the UV light (Figure 5C). The supernatants of homogenized pNMV-hPAP1 and pNMV-hGFP expressing leaves were markedly different in color (Figure 5D), with the orange color indicating the production of anthocyanins. Accumulation of delphinidin-based anthocyanins in the pNMV-hPAP1 plants was confirmed by TLC (Figure 5E). The pPAP1-GFP vector also generated colored foci, but with a much reduced efficiency than pNMV-hPAP1 (data not shown).

### Changes in flavonoid production resulting from PAP1and PAP1-GFP expression in *N. benthamiana* leaves

**Figure 6:**
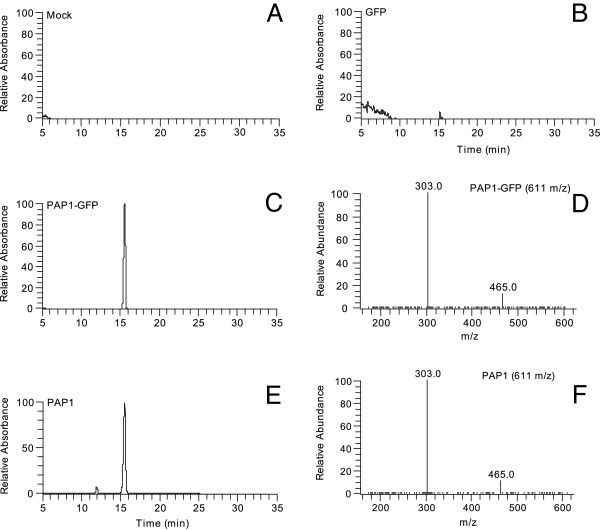
**LC/MS Anthocyanin analyses in the leaves of *N. benthamiana *plants with different inoculations.** LC peaks were revealed at 520 nm absorption wavelength. **A**: LC, mock-inoculated leaves; **B**: LC, the leaves inoculated with viruses derived from pNMV-GFP; **C**: LC, the leaves inoculated with viruses derived from pPAP1-GFP; **D**: MS fragmentation of the molecular ion of delphinidin-3-*O*-rutinoside (*m/z* 611) in the peak detected at 520 nm in **C**; **E**: LC, the leaves inoculated with viruses derived from pNMV-PAP1; **F**: MS fragmentation of the molecular ion (611 m/z) in the peak detected at 520 nm in E to reveal the component ions delphinidin-3-*O*-glucoside (*m/z* 465) and delphinidin (*m/z* 303).

To investigate the effect of pNMV-expressed PAP1 and PAP1-GFP proteins on the flavonoid pathways in the *N. benthamiana* leaves, flavonoid metabolic profiles of leaves inoculated with extracts for PAP1, PAP1-GFP, GFP and the mock control were analyzed using LC-MS. The LC results showed no detectable anthocyanins in the leaves of the mock control or plants expressing GFP only (Figures 6A and B). However, the leaves expressing PAP1-GFP or PAP1 produced an identical anthocyanin peak with an absorption at 525 nm (Figures 6C-F), in agreement with the visual observation and TLC results (Figure 5E). This anthocyanin peak generated a molecular ion mass at m/z 611, suggesting that the anthocyanin was delphinidin-3-*O*-rutinoside (a glucose at the C-3, with a rhamnose attached to the glucose). This was verified by further fragmentation of m/z 611 into m/z 303 (delphinidin), m/z 465 (delphinidin-3-*O*-glucoside) and the m/z 144 (rhamnose) (Figures 6D, F).

**Figure 7:**
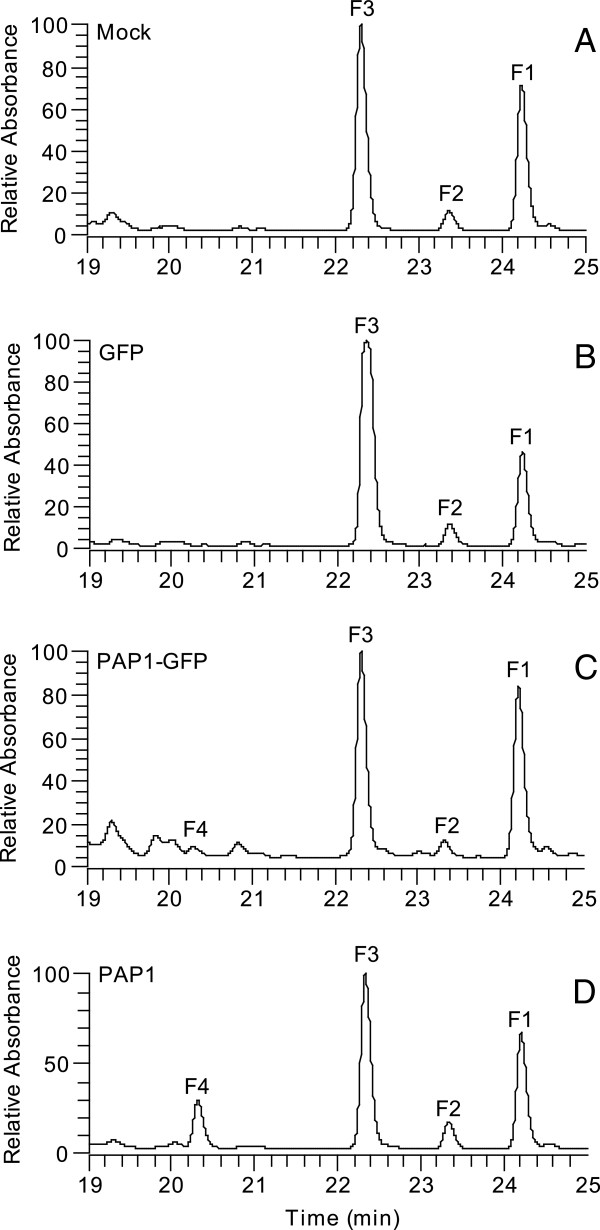
**LC Profile of flavonoids in the leaves of *N. benthamiana *plants with different inoculations.** The LC peaks were detected at 320 nm absorption wavelength. **A**: mock-inoculated leaves; **B**: the leaves inoculated with viruses derived from pNMV-GFP; **C**: the leaves inoculated with viruses derived from pPAP1-GFP; **D**: the leaves inoculated with viruses derived from pNMV-PAP1. F1, kaempferol-3-*O*-rutinoside, F2 quercetin-3-*O*-glucoside, F3, quercetin-3-*O*-rutinoside and F4, myricetin-3-*O*-rutinoside.

Although *N. benthamiana* leaves do not produce anthocyanins naturally, they are capable of producing another group of flavonoids, the colorless flavonols. LC results showed that three flavonoid peaks were detected in the mock control and the leaves expressing GFP, and the patterns of the peaks appeared almost identical (Figures 7A and B), indicating that viral activity alone did not alter the flavonol profile. However, leaves expressing PAP1 or PAP1-GFP produced not only the three peaks present in control plants (F1, F2 and F3) but also a new peak, F4 (Figures 7C and D). Peak F4 was much higher in the leaves expressing PAP1 than in those expressing PAP1-GFP. MS analysis revealed that in all the samples the peaks for F1, F2 and F3 produced molecular ion masses at m/z 595, 465 and 611, respectively (Additional file 4: Figure S3). Further fragmentation indicated that all three compounds were flavonols, with m/z 595 (F1) being kaempferol-3-*O*-rutinoside (m/z 287 and m/z 449), m/z 465 (F2) being quercetin-3-*O*-glucoside (m/z 303) and m/z 611 (F3) being quercetin-3-*O*-rutinoside (m/z 303 and m/z 465). Peak F4 had a molecular ion mass at m/z 627, and no trace of ion mass at m/z 627 were detected in mock or pNMV-hGFP-inoculated control plants. Mass fragmentation indicated that m/z 627 represented the flavonol myricetin-3-*O*-rutinoside (m/z 319 and m/z 481). Myricetin has three hydroxyl groups on the B-ring of the molecular (C-3′, 4′ and 5′), while quercetin and kaempferol have only two or one B-ring hydroxyl, respectively.

### Efficiency of NMV replication in *N. benthamiana*

**Figure 8:**
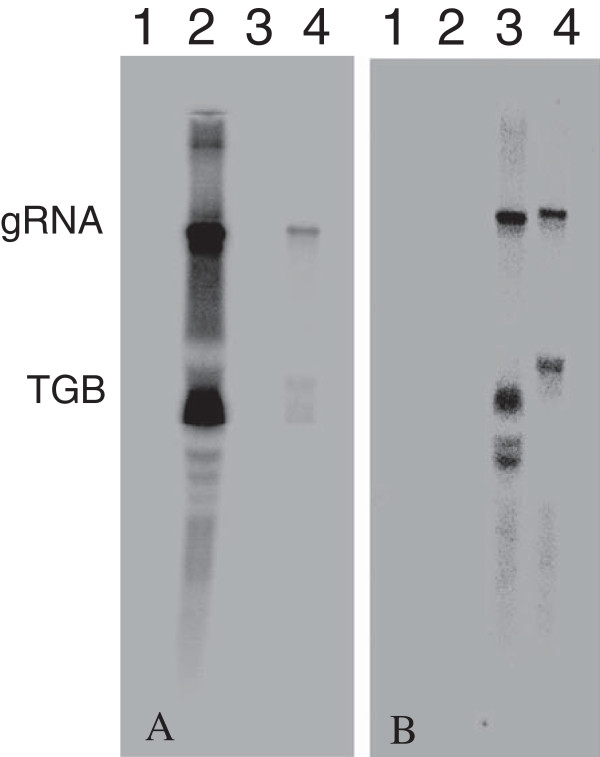
**Northern blot for viral replication analyses.** Total RNAs (20.0 μg) for each treatment were separated on 1.2% agarose gel, transferred onto nylon membrane and probed with respective radiolabeled DNA probes. **A**: Northern blot probed with radiolabeled GFP DNA probe. **B**: Northern blot probed with radiolabeled *PAP1* DNA probe. Lane 1, pNMV-hGFP inoculation, Lane 2: pNMV-hPAP1 inoculation; Lane 3, pNMV-PAP1-GFP inoculation.

Northern RNA analysis with GFP and PAP1 cDNA probes was used to assess the genome replication of the NMV vectors in *N. benthamiana*. Both probes revealed the genomic and triple gene block subgenomic RNAs but not the region corresponding to the CP sequence (Figure 8). With GFP probe, the RNA genome of viruses from pNMV-hGFP accumulated to high amounts in the leaves (Figure 8A). However, while the RNA genome of viruses derived from pPAP1-GFP was easily detected, the concentrations were much lower than those from pNMV-hGFP (Figure 8A). The mock control and samples from pNMV-hPAP1 inoculation did not produce any signal with the GFP probe, as expected. When the PAP1 probe was used, both genomes of transgenic viruses from pNMV-hPAP1 and pPAP1-GFP were detected in the *N. benthamiana* leaves, although again at lower concentrations than when pPAP1-GFP vector was used (Figure 8B). Based on the size detected, full-length viral genomic RNAs were accumulated (Figure 8).

## Discussion

A range of virus-based plant transient expression systems has been developed for protein expression [13,16,35-39]. Plant viral vector systems have several advantages over other systems, such as rapid and systemic infections, and also avoid the risks associated with the use of animal pathogens for production of recombinant proteins or vaccines [11,40-43]. However, there are also drawback of viral-based systems, including pathogenic host responses, spread to non-target plants and a lack of viral systems for a wide range of host plants. We report here a new viral expression system based on NMV and *N. benthamiana*, and incorporating the Gateway® cloning system, that addresses some of these drawbacks.

NMV isolated from New Zealand is a mild strain that replicates very efficiently in *N. benthamiana* but does not cause visible pathogenic symptoms. It also has a very limited host range, reducing the risk of the unintentional spread of the virus. *N. benthamiana* is a model species of growing importance [22,44], and has previously been used with viral vectors for studies of secondary metabolism, such as alkaloid biosynthesis [45], the production of flavor enzymes [46], and as a production platform for small pharmaceutical molecules [47].

The Gateway® system was incorporated into the base NMV vectors we developed to enable higher throughput cloning of the genes of interest without the need for use of restriction enzymes. Gateway® technology is well established as a highly efficient system for high throughput applications [37,48-52]. The effectiveness of the base NMV vectors (pNMV-GW, pNMV-GWg and pNMV-hGW) for Gateway® cloning and protein expression was demonstrated for both reporter genes and genes involved in the regulation of endogenous biosynthetic pathways. The base vectors were all shown to drive accumulation of expressed protein after only a few days post-inoculation, using either biolistic DNA delivery or a viral particle inoculation. The inclusion of a His-tag enabls rapid protein purification. A simple Ni-affinity purification step led to the highly purified GUS protein, as evidenced by commassie blue staining and the Western blot analysis. The purified level of GUS protein by Ni-affinity (10 mg per kg FW) suggests that the NMV viral system has the potential to produce high level proteins, given the material used for protein measurement only had local infection foci (Additional file 1: Figure S1) on the biolistically transformed leaf. The local foci account for a very small proportion of the leaf tissue (systemic infection would produce much higher proportion of infected materials). As an alternative to the His-tag, the DEAE column tests and Western blotting analyses with GUS suggested that NMV-expressed proteins can be easily enriched by column chromatography while retaining protein functionality.

The effect of the His-tag on the GFP for determining sub-cellular localization of proteins was examined in detail using GFP with or without ER retaining signals. Clearly, addition of the His-tag to both versions of the GFP did not alter the behavior of intracellular trafficking of the GFP. The results indicates that the His-tag will be unlikely to affect the function of a expressed protein, as confirmed in this study, where the His-tagged PAP1 was highly efficient in turning on the limiting step(s) of anthocyanin biosynthesis pathway in *N. benthamiana,* a species that is normally anthocyanin-less. A further advantage of the NMV vectors for localization studies is that infection foci are easily visible as GFP-positive foci, because of multi-celled infection clusters forming, but within the foci, cells showing a range of expression levels of the GFP-tagged protein are apparent, allowing studies as to whether the protein concentration is altering localization.

Higher protein concentrations were achievable when an ER retention signal was added to the vectors, although this by nature precludes studies on normal sub-cellular localization. The higher GFP signals seen when using the ER signal motifs support previous studies on ER retention as a means to increase protein over-expression levels [30]. ER retaining signals can also be used to alter the post translational modification of an expressed protein [53]. The Western analyses in this study has suggested that the His-tag along with the N-terminal ER signal were removed in the plant cell after the protein was transported into ER, according to the Western analyses for the total protein. This was confirmed with Ni-affinity purification of the GFPs, as the GFP tagged with both His and ER signal peptide was unable to be retained by the Ni-affinity material. However, the very small His-tag could be placed behind the N-terminal ER signal or C-terminus of the fused proteins to prevent its being cleaved.

Transgenic viruses often show instability of the inserted protein sequence [35], particularly with large sequence inserts. However, the NMV system was effective in expressing both relatively small (GFP) and large (GUS) proteins within the timeframe of the experiments carried out in this report. The results in this study also indicated that gene inserts in the NMV viral vectors are stable after the passage from the biolistically transformed material to intact plants, as evidenced by the efficient expression of the insert genes in the whole plant. The capability to express GUS protein (68 KDa) in a NMV system suggests that large transcription factors, such as bHLHs that are involved in the regulation of flavonoid biosynthesis pathways, could also be expressed with this system. The pattern of GFP spread (locally and systemically) found in this study suggests that NMV shows behaviors similar to those characterized for other Potex viruses [16,54,55]. This long period of stable protein expression, in combination with the ease of systemic infection, suggests that the NMV system may provide an efficient platform for over-expression, purification and sub-cellular localization of proteins of interest.

The results show that NMV vectors can be used in the expression and purification of proteins *in planta*. The applicability of the NMV system for studying the function of expressed proteins in endogenous metabolic pathways was demonstrated by using the anthocyanin pathway-related R2R3 MYB PAP1 (AtMYB75). *N. benthamiana* has been demonstrated to be an effective model plant for identifying proteins associated with secondary metabolism [45,56,57]. However, for unknown reasons, the laboratory lines of *N. benthamiana* used do not show production of anthocyanin pigments. PAP1 is a strong activator of anthocyanin biosynthesis in both *Arabidopsis* and heterologous hosts [33], and it was decided to examine whether NMV-based over-expression of PAP1 could induce anthocyanin production in *N. benthamiana* leaves.

Dark-red anthocyanic-like foci were clearly visible by 7 dpi of the His-tagged PAP1 virion on the leaves of intact plants, suggesting that the entire anthocyanin biosynthetic pathway is present in *N. benthamiana*, and that lack of activity of a regulatory gene expression is the cause of the usual acyanic phenotype. The inclusion of the His-tag or a GFP fusion did not prevent PAP1 activity, suggesting they did not interfere with the formation of the necessary MYB-bHLH-WD40 anthocyanin regulatory complex in plants [28,58-60]. This is despite the fact that the GFP protein of 27 kDa is of a similar size to the MYB protein (approximately 28 kDa). Some viruses can be responsible for anthocyanin accumulation in other plant species [61]; however, the control of NMV expressing GFP and mock-inoculation controls did not show any anthocyanin induction.

LC/MS was used to confirm that anthocyanin production was indeed induced by PAP1 over-expression, with delphinidin-3-*O*-rutinoside found to accumulate. It also allowed for the examination of the effect of PAP1 on non-anthocyanin flavonoids. Although control *N. benthamiana* plants lacked anthocyanins, they had a basal production of the flavonols kaempferol-3-*O*-rutinoside and quercetin-3-*O*-rutinoside, which are from branches off the flavonoid pathway prior to anthocyanins. Interestingly, in addition to inducing anthocyanin production, PAP1 and PAP1-GFP over-expression also caused the accumulation of a flavonol not detected in the control plants, myricetin-3-*O*-rutinoside. As both delphinidin and myricetin have 3′4′5′-hydroxylation of the flavonoid B-ring, this suggests that PAP1 is activating the gene(s) for the flavonoid 3′5′-hydroxylase (F3′5′H). Interestingly, one of the reasons behind high anthocyanin production in tomato only being possible with specific anthocyanin-related transcription factor combinations is thought to be whether they can activate the F3′5′H, as specificity of substrate use is known for some of the flavonoid biosynthetic enzymes in the Solanaceae [62].

Based on RNA analysis, there was variation in the replication efficiency of the vectors. The GFP-containing NMV replicated more efficiently than either the PAP1 vector, which is a DNA insert of similar length to GFP, or the PAP1-GFP vector. The RNA measurements match visual observations that GFP foci appear earlier on inoculated leaves when using the GFP vector. One possibility is that the slow movement or replication of the PAP1 containing NMV was caused by the very high concentrations of the flavonoids accumulated in the cells, as flavonoids have been suggested to have anti-viral roles [63].

In conclusion, the results presented here demonstrate that the combination of the NMV Gateway vectors and *N. benthamiana* provides an efficient platform for protein expression, purification, intracellular localization and also plant secondary metabolite production, as a research tool and also potentially as a biotechnology tool. A recent published work [64] has reinforced the value of plant viral vectors in not only the production of macromolecules but also small phytochemicals such as plant pigments.

## Authors’ contributions

HZ designed, carried out the experiments and wrote the manuscript draft. LW contributed to the vector cloning and the Northern analysis. DH contributed to the vector cloning, result analysis and the writing of the manuscript. CV made the cDNA clone of the Narcissus Mosaic virus. NJ carried out the LC-MS KD contributed to research management, result analysis and the writing of the manuscript. All authors and approved the final manuscript.

## Supplementary Material

Additional file 1: Table S1 Primers used in constructing the *attB1* and *attB2* flanked PCR products for *pNMV-hGUS, pNMV-hGFP, pNMV-hGFPer, pNMV-PAP1-GFP, pNMV-SeBP-GFP pNMV-hPAP1*. High fidelity PWO DNA polymerase (Roche) was used. Click here for file

Additional file 2: Figure S1 Detached *Nicotiana benthamiana* leaves incubated for 5 days in the dark after biolistic introduction of NMV-hGUS DNAs. The GUS staining was according to the method of [32]. Click here for file

Additional file 3: Figure S2 Detached *Nicotiana benthamiana* leaves incubated for 5 days in the dark after biolistic introduction of pNMV-hGFP and pNMV-hPAP1 DNAs. Click here for file

Additional file 4: Figure S3 MS fragmentation of the flavonoids revealed in the Figure 7. F1, kaempferol-3-*O*-rutinoside (*m/z* 595), kaempferol-3-*O*-glucoside (*m/z* 449), kaempferol (*m/z* 287); F2, quercetin-3-*O*-glucoside (*m/z* 465), quercetin (*m/z* 303) , F3, quercetin-3-*O*-rutinoside (*m/z* 611), quercetin-3-*O*-glucoside (*m/z* 465), quercetin (*m/z* 303) and F4, myricetin-3-*O*-rutinoside (*m/z* 627), myricetin-3-*O*-glucoside (*m/z* 481) and myricetin (*m/z* 319). Click here for file
