# Supplementary material for: A Narcissus mosaic viral vector system for protein expression and flavonoid production
Source: Plant Methods. 2013 Jul 13;9:28. doi: 10.1186/1746-4811-9-28 (PMC3728148; doi:10.1186/1746-4811-9-28)
Supplement: Additional file 1: Table S1 — Primers used in constructing the attB1 and attB2 flanked PCR products for pNMV-hGUS, pNMV-hGFP, pNMV-hGFPer, pNMV-PAP1-GFP, pNMV-SeBP-GFP pNMV-hPAP1. High fidelity PWO DNA polymerase (Roche) was used. [file 1746-4811-9-28-S1.docx]

| Primers | Forward | Reverse |
| --- | --- | --- |
| *GUS* | attB1-TTACGTCCTGTAGAAACCCCAA | attB2-TCATTGTTTGCCTCCCTGCTG |
| *GFP* | attB1-ATGAGTAAAGGAGGAGGACTTTTC | attB2-TTATTTGTATAGTTCATCCATGCCA |
| *GFPer* | aatB1-ATGAAGACTAATCTTTTTCTCTTTC | attB2-TTAAAGCTCATCATGTTTGATTAG |
| *PAP1(-GFP)* | attB1-ATGGAGGGTTCGTCCAAAGG | AttB2-CCATCAAATTTCACAGTCTCTC |
| *SeBP(-GFP)* | attB1-ATGGCAACCGAAACCGTATTAG | attB2-CCGATCCAGATATCGGAAGTG |
| *PAP1* | attB1-ATGGAGGGTTCGTCCAAAGG | attB2-CCTATACACAAACGCAAACAAA |

Table S1: Primers used in constructing the *attB1* and *attB2* flanked PCR products for *pNMV-hGUS, pNMV-hGFP, pNMV-hGFPer, pNMV-PAP1-GFP, pNMV-SeBP-GFP pNMV-hPAP1*. High fidelity PWO DNA polymerase (Roche) was used.
