# Supplementary material for: A Narcissus mosaic viral vector system for protein expression and flavonoid production
Source: Plant Methods. 2013 Jul 13;9:28. doi: 10.1186/1746-4811-9-28 (PMC3728148; doi:10.1186/1746-4811-9-28)
Supplement: Additional file 2: Figure S1 — Detached Nicotiana benthamiana leaves incubated for 5 days in the dark after biolistic introduction of NMV-hGUS DNAs. The GUS staining was according to the method of [32]. [file 1746-4811-9-28-S2.pdf]

pNMV-hGUS, 5 dpi

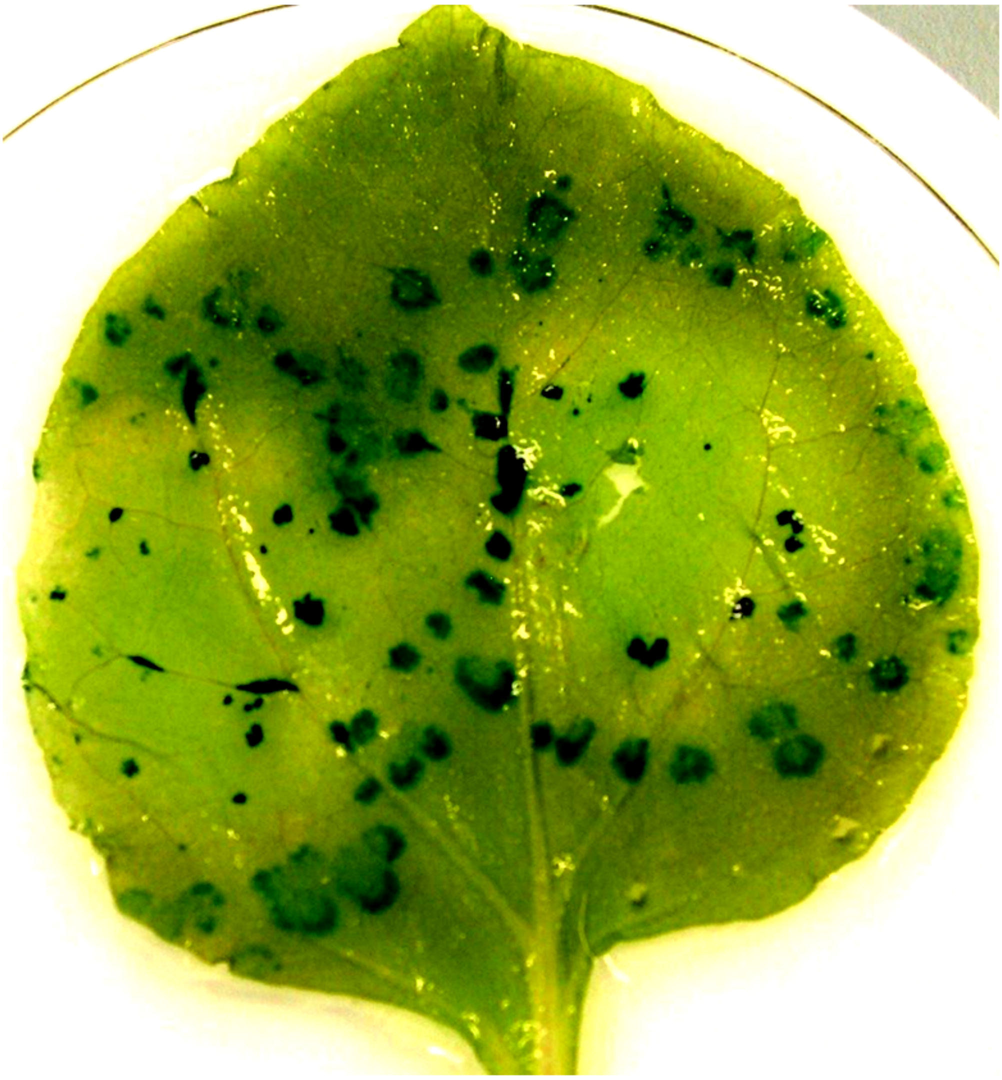

S1: Detached *Nicotiana benthamiana* leaves incubated for 5 days in the dark after biolistic introduction of NMV-hGUS DNAs. The GUS staining was according to the method of (Shang et al., 2007).
