# Supplementary material for: A Narcissus mosaic viral vector system for protein expression and flavonoid production
Source: Plant Methods. 2013 Jul 13;9:28. doi: 10.1186/1746-4811-9-28 (PMC3728148; doi:10.1186/1746-4811-9-28)
Supplement: Additional file 3: Figure S2 — Detached Nicotiana benthamiana leaves incubated for 5 days in the dark after biolistic introduction of pNMV-hGFP and pNMV-hPAP1 DNAs. [file 1746-4811-9-28-S3.pdf]

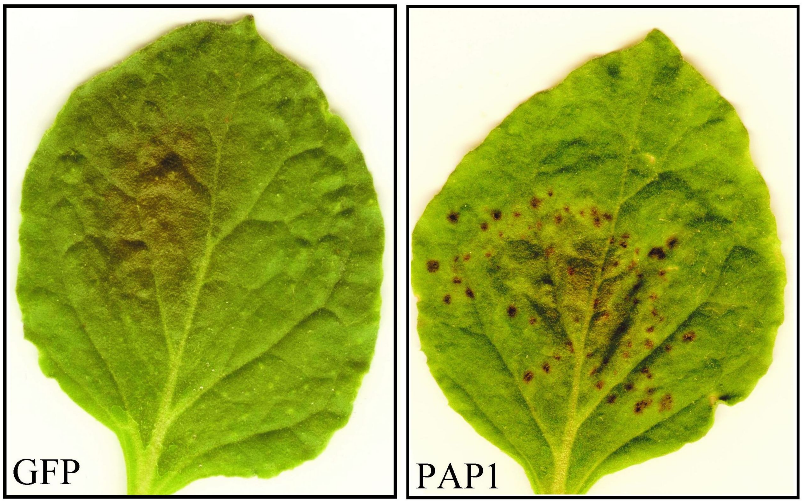

S2: Detached *Nicotiana benthamiana* leaves incubated for 5 days in the dark after biolistic introduction of pNMV-hGFP and pNMV-hPAP1 DNAs.
