# Supplementary material for: A Narcissus mosaic viral vector system for protein expression and flavonoid production
Source: Plant Methods. 2013 Jul 13;9:28. doi: 10.1186/1746-4811-9-28 (PMC3728148; doi:10.1186/1746-4811-9-28)
Supplement: Additional file 4: Figure S3 — MS fragmentation of the flavonoids revealed in the Figure 7. F1, kaempferol-3-O-rutinoside (m/z 595), kaempferol-3-O-glucoside (m/z 449), kaempferol (m/z 287); F2, quercetin-3-O-glucoside (m/z 465), quercetin (m/z 303) , F3, quercetin-3-O-rutinoside (m/z 611), quercetin-3-O-glucoside (m/z 465), quercetin (m/z 303) and F4, myricetin-3-O-rutinoside (m/z 627), myricetin-3-O-glucoside (m/z 481) and myricetin (m/z 319). [file 1746-4811-9-28-S4.pdf]

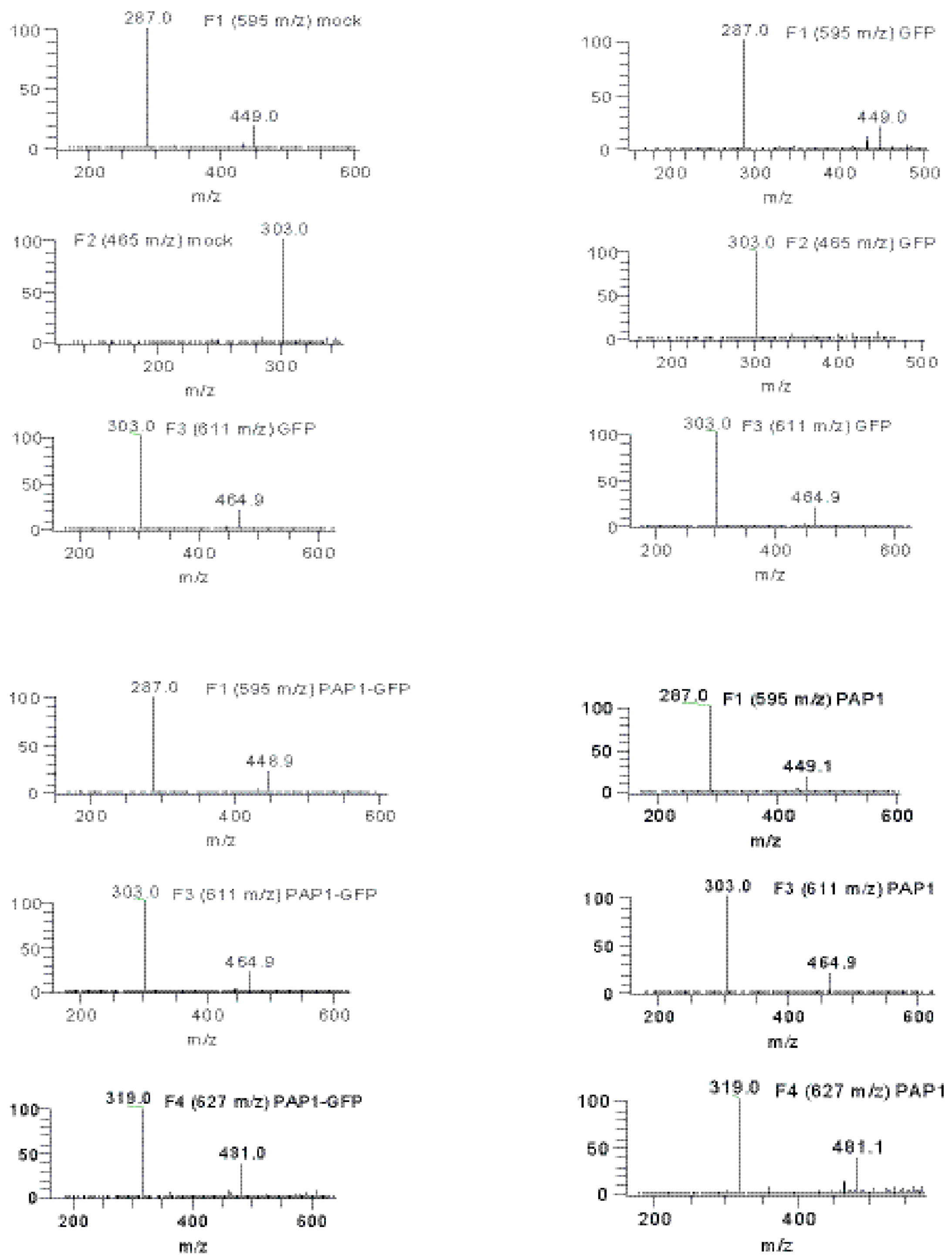


S3: MS fragmentation of the flavonoids revealed in the Figure 7. F1, kaempferol-3-*O*-rutinoside (*m/z* 595), kaempferol-3-*O*-glucoside (*m/z* 449), kaempferol (*m/z* 287); F2, quercetin-3-*O*-glucoside (*m/z* 465), quercetin (*m/z* 303) , F3, quercetin-3-*O*-rutinoside (*m/z* 611), quercetin-3-*O*-glucoside (*m/z* 465), quercetin (*m/z* 303) and F4, myricetin-3-*O*-rutinoside (*m/z* 627), myricetin-3-*O*-glucoside (*m/z* 481) and myricetin (*m/z* 319).
